# Supplementary material for: Helix 8 is the essential structural motif of mechanosensitive GPCRs
Source: Nat Commun. 2019 Dec 19;10:5784. doi: 10.1038/s41467-019-13722-0 (PMC6923424; doi:10.1038/s41467-019-13722-0)
Supplement: Supplementary file 1 — Supplementary Information [file 41467_2019_13722_MOESM1_ESM.pdf]

Helix 8 is the essential structural motif of mechanosensitive GPCRs

Erdogmus et al.

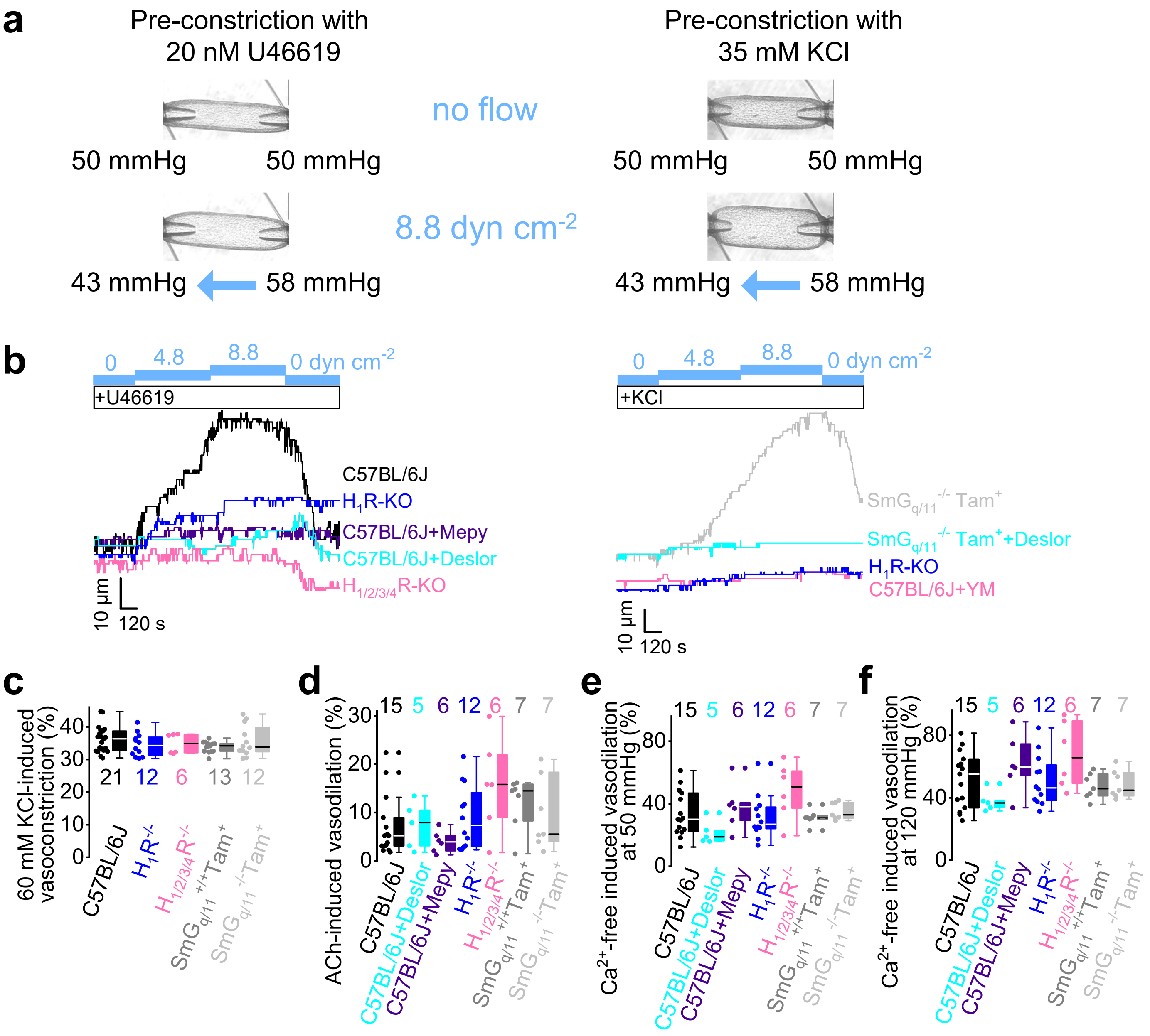

Supplementary Figure 1. Measurements of flow-induced vasodilation. **a** Representative pictures of mesentery artery segments of first and second order branches in the presence of 20 nM U46619 (left) or 35 mM KCl (right) displayed at no flow conditions (inflow and outflow pressure was 50 mmHg) and at intravascular flow of Δ15 mmHg (inflow pressure was 58 mmHg and outflow pressure was 43 mmHg) which corresponds to 8.8 dyn cm^{-2}. Blue arrows indicate the flow direction. **b** Representative original time courses of vessel outer diameters of mesentery artery segments (resolution of 1 µm). Vessels from wild-type mice (C57BL/6J), from tamoxifen-treated, smooth muscle specific $G_{q/11}$-knock-down mice ($SmG_{q/11}{}^{-/-}$ Tam^+), from H_1R gene-deficient mice ($H_1R^{-/-}$) and of $H_1/H_2/H_3/H_4R$ quadruple gene-deficient mice ($H_{1/2/3/4}R^{-/-}$). Flow rates corresponded to shear stress of 4.8±0.5 dyn cm^{-2} and 8.8±1.1 dyn cm^{-2}. The inverse H_1R agonists mepyramine (100 µM, Mepy) and desloratadine (30 µM, Deslor) and the selective $G_{q/11}$-protein inhibitor YM254890 (100 nM, YM) were intravascularly applied and incubated for 30 minutes (related to Figure 1). **c-f** Parameters of analyzed mesentery artery segments. **c** Maximal vasoconstriction by 60 mM KCl. **d** Vasodilation by 300 µM acetylcholine (ACh). **e**, **f** Maximal vasodilation by Ca^{2+}-free solution at intraluminal pressure of 50 mmHg (**e**) and at 120 mmHg (**f**). No significant differences were observed using Kruskal-Wallis test. Data are presented as boxplots with means plus interquartile ranges. Whiskers indicate 1.5fold interquartile range. Related to Figure 1.

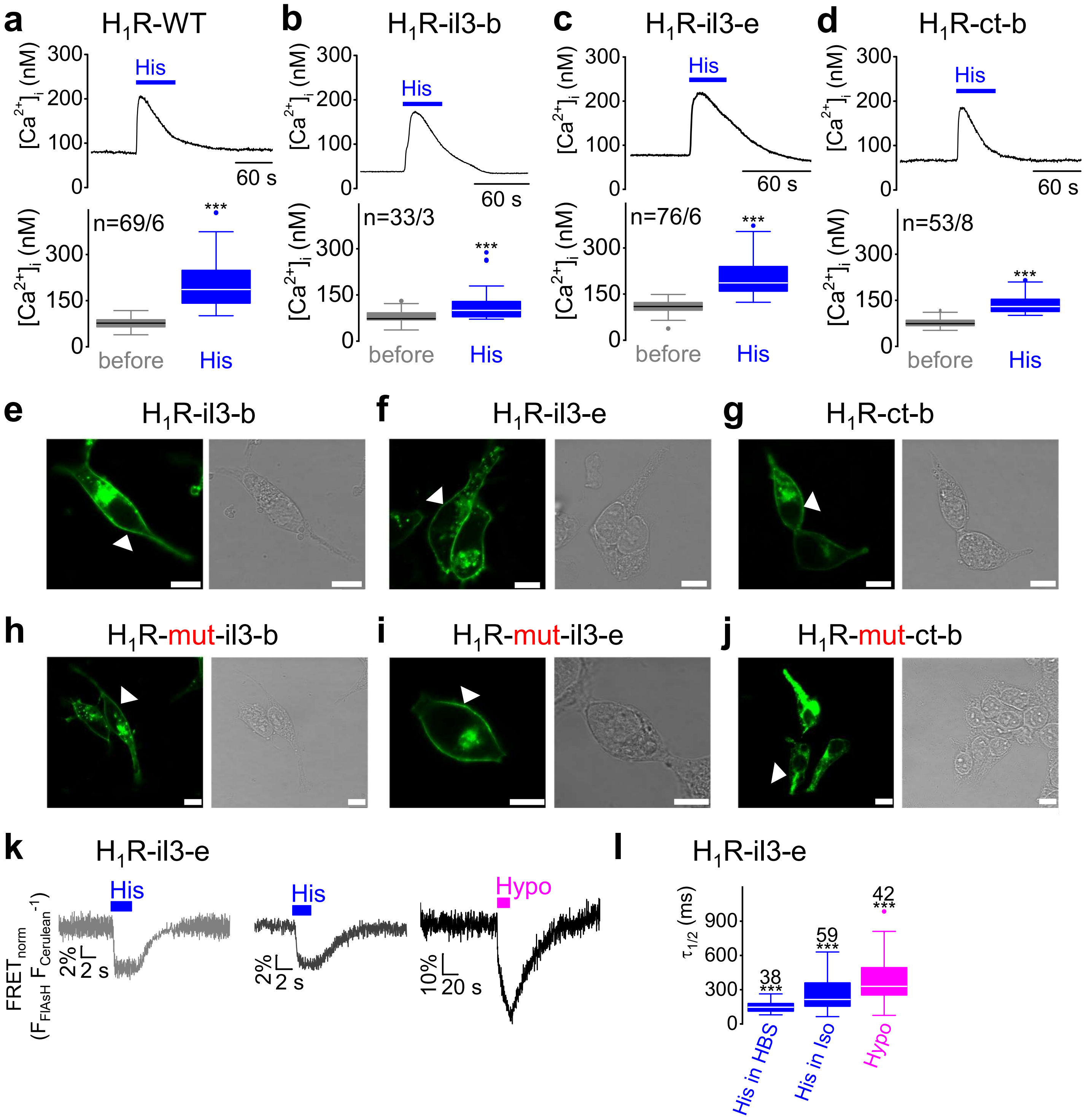

Supplementary Figure 2. H_1R FRET constructs are functional and agonist- and hypoosmotically induced FRET signals exhibit distinct kinetics. a-d Calcium imaging of HEK293 cells expressing gpH_1R (H_1R-WT) or the FRET constructs H_1R-il3-b, H_1R-il3-e and H_1R-ct-b. Representative traces $[Ca^{2+}]_i$ (above) with application of 100 µM histamine (His). Summaries of $[Ca^{2+}]_i$ before and during application of histamine (below). n = x/y indicates the sample size, where x is the number of measured cells and y is the number of coverslips from at least 3 experimental days. ****P*<0.001; Wilcoxon matched-pairs signed-rank test to compare to basal $[Ca^{2+}]_i$ before histamine application. **e-j** Representative confocal images of HEK293 cells expressing indicated FRET constructs showing membrane staining of the cerulean fluorescence (indicated with white arrows). Scale bars indicate 10 µm. **k**, **l** FRET measurements with the H_1R-il3-e FRET construct. **k** Representative FRET traces with application of histamine 'His' and of hypoosmotic bath solution (Hypo). Histamine was applied in physiological (HBS) (left) or in isotonic bath solution supplemented with mannitol (Iso) (middle). Hypoosmotic solution was applied subsequent to isotonic bath solution (right). **l** Summary of the kinetics of FRET signal decreases. Numbers indicate the numbers of measured cells from at least 3 experimental days. ****P*<0.001; Kruskal-Wallis test. Data are presented as boxplots with means plus interquartile ranges. Whiskers indicate 1.5fold interquartile range. Related to Figure 2 and Figure 3.

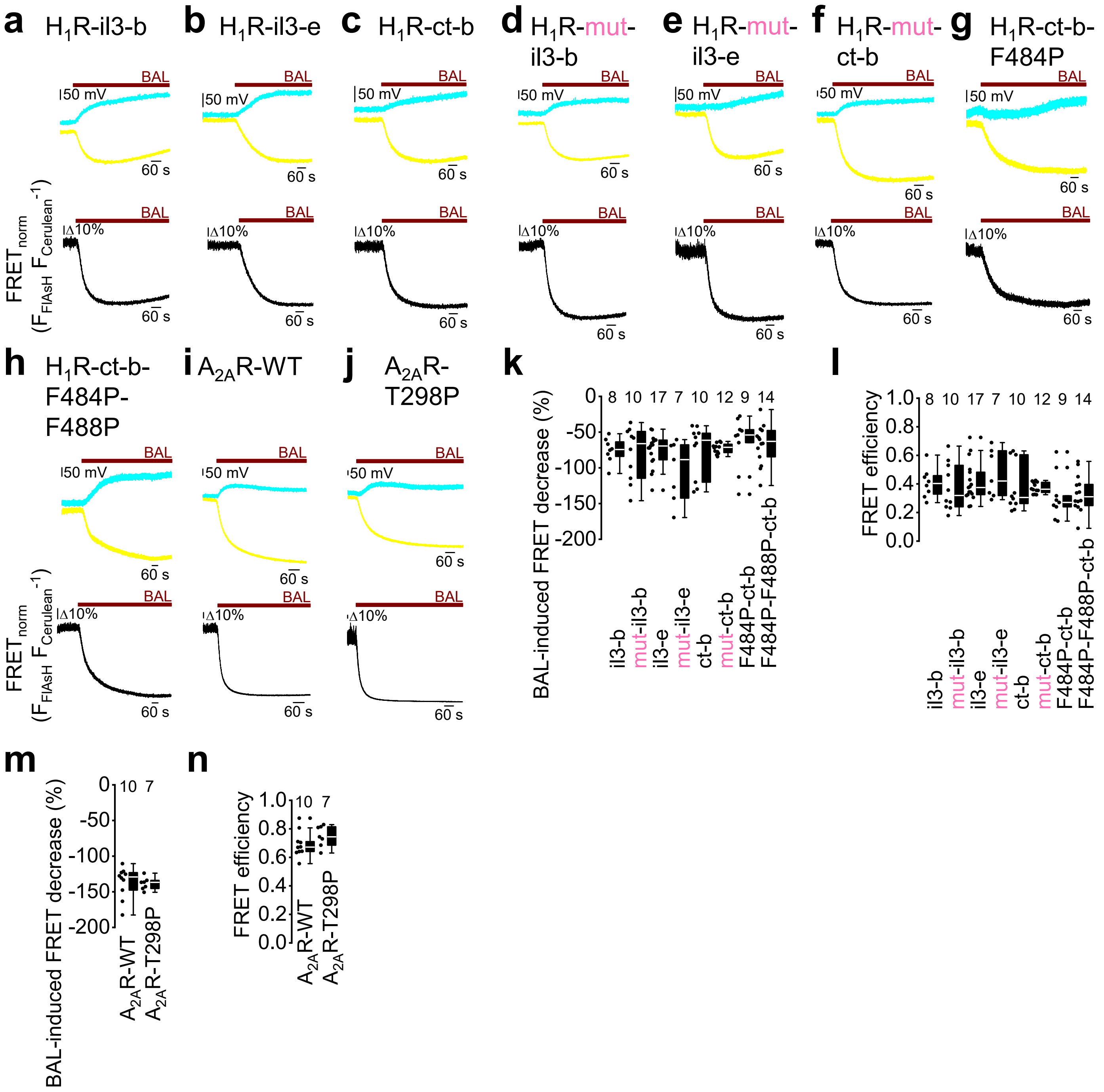

Supplementary Figure 3. BAL-induced FRET signal decreases and FRET efficiencies are comparable. a-n Representative FRET measurements of indicated FlAsH-labeled H_1R and $A_{2A}R$ FRET constructs H_1R-il3-b (**a**), H_1R-il3-e (**b**), H_1R-ct-b (**c**), of the histamine binding site mutants H_1R-mut-il3-b, H_1R-mut-il3-e and H_1R-mut-ct-b (**d-f**), of the H_1R-ct-b constructs with disrupted H8 by point mutations H_1R-ct-b-F484P and H_1R-ct-b-F484P-F488P (**g** and **h**), of the $A_{2A}R$ FRET construct $A_{2A}R$-WT (**i**) and of the $A_{2A}R$ FRET construct with disruption of H8 $A_{2A}R$-T298P (**j**). 10 mM BAL was applied to quench FlAsH fluorescence and to dequench cerulean fluorescence. Traces of the cerulean (cyan) and of the FlAsH fluorescence (yellow) measured as voltage of the transimpedance amplifier are displayed above. Black traces show normalized FRET signals determined as ratio of the normalized FlAsH and cerulean fluorescences. **k-n** Summaries of BAL-induced FRET signal changes (**k** and **m**) and calculated FRET efficiencies (**l** and **n**) are presented as boxplots with means plus interquartile ranges. Whiskers indicate 1.5fold interquartile range. Numbers over boxes indicate numbers of measured cells from at least 3 experimental days. There were no significant differences between the eight constructs tested with Kruskal-Wallis test. Related to Figure 2, Figure 3, Figure 4 and Figure 5.

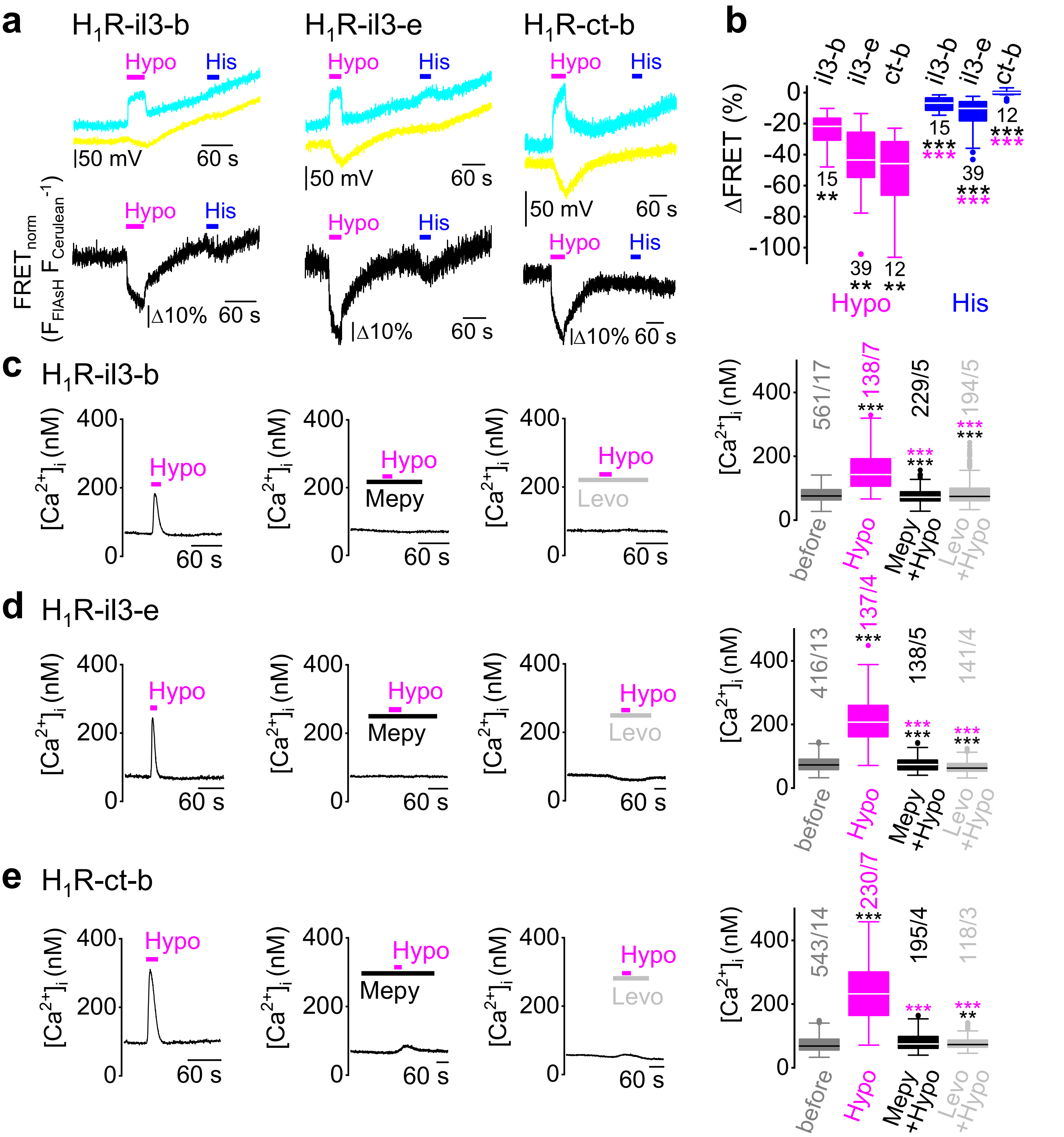

Supplementary Figure 4. Distinct agonist and membrane stretch-induced active receptor conformations. a Representative FRET measurements with the FlAsH-labeled FRET constructs H_1R-il3-b, H_1R-il3-e and H_1R-ct-b. Applications of hypoosmotic solution (Hypo, 150 mOsm kg^{-1}) and of 100 μM histamine (His). Traces of the cerulean (cyan) and of the FlAsH fluorescence (yellow) measured as voltage of the transimpedance amplifier. Black traces show normalized FRET signals **b** Summary of membrane stretch- and histamine-induced FRET signal changes. Numbers indicate the number of measured cells from at least 3 experimental days. $^{**}P<0.01$, $^{***}P<0.001$, black asterisks; Kruskal-Wallis test and $^{***}P<0.001$, magenta asterisks; Wilcoxon matched-pairs signed-rank test to compare between membrane stretch- and histamine-induced FRET signals of each construct. **c-e** Calcium imaging with HEK293 cells stably expressing indicated H_1R-FRET constructs. Representative traces of $[Ca^{2+}]_i$ with application of hypoosmotic solution (Hypo) in the presence and absence of 30 μM mepyramine (Mepy) and 10 μM levocetirizine (Levo) (left). Summaries of $[Ca^{2+}]_i$ (right). $^{***}P<0.001$, black asterisks; matched-pairs signed-rank test to compare to basal $[Ca^{2+}]_i$ using, and $^{**}P<0.01$ and $^{***}P<0.001$, magenta asterisks; Mann-Whitney *U* test to compare to hypoosmotically induced $[Ca^{2+}]_i$ (**a**, **b**). n = x/y indicates the sample size, where x is the number of measured cells and y is the number of coverslips from at least 3 experimental days. **b**, **c-e** Data are presented as boxplots with means plus interquartile ranges. Whiskers indicate 1.5fold interquartile range Related to Figure 2.

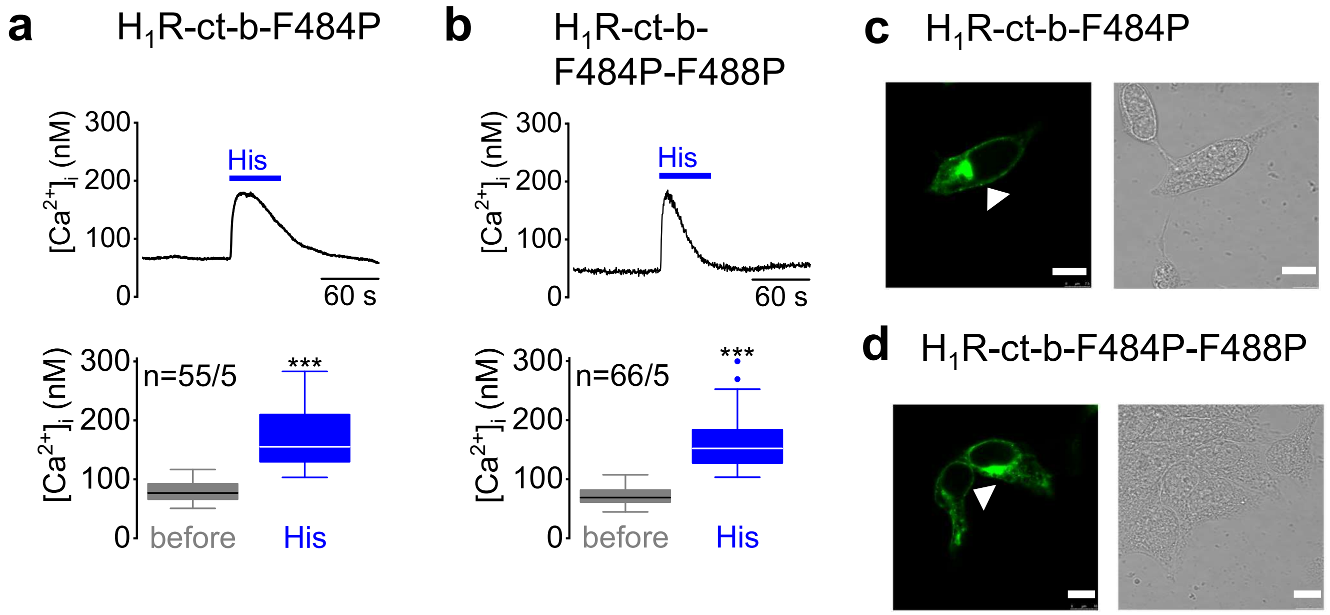

Supplementary Figure 5. H8 mutants of H_1R are functionally expressed and show agonist-induced calcium increases. a, **b** Calcium imaging with HEK293 cells transiently transfected with the FRET constructs H_1R-ct-b-F484P (**a**) and H_1R-ct-b-F484P-F488P (**b**). Representative traces of $[Ca^{2+}]_i$ (above) with indicated application of 100 µM histamine (His). Summaries of $[Ca^{2+}]_i$ before and during application of histamine are shown below. n = x/y indicates the sample size, where x is the number of measured cells and y is the number of coverslips from at least 3 experimental days. ***P<0.001, black asterisks; Wilcoxon matched-pairs signed-rank test to compare to basal $[Ca^{2+}]_i$ before histamine application. Data are presented as boxplots with means plus interquartile ranges. Whiskers indicate 1.5fold interquartile range. **c**, **d** Representative confocal images of HEK293 cells transfected with the FRET constructs H_1R-ct-b-F484P (**c**) and H_1R-ct-b-F484P-F488P (**d**) showing membrane staining of cerulean fluorescence (indicated with white arrows). Scale bars indicate 10 µm. Related to Figure 4.

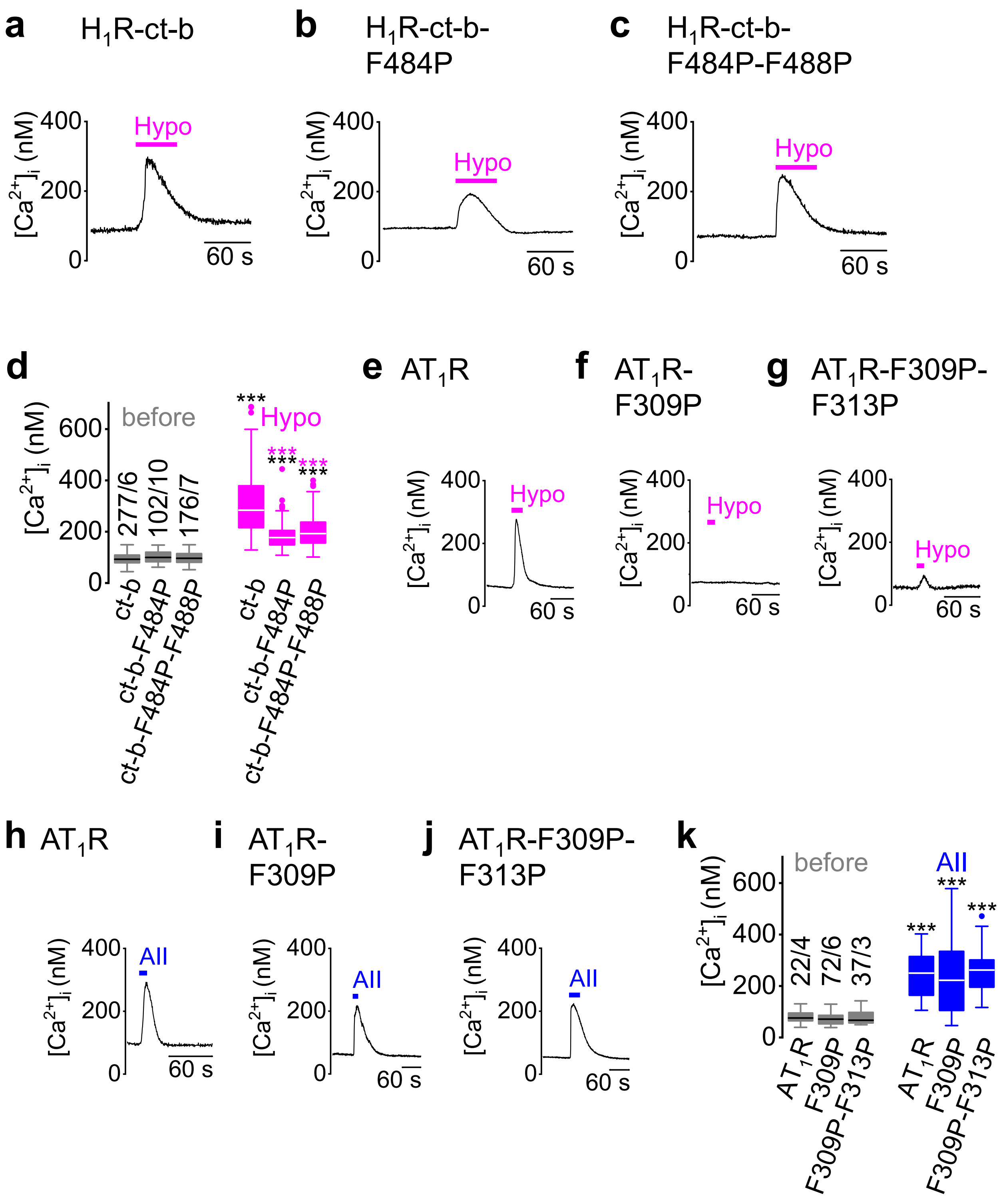

Supplementary Figure 6. H8 mutants show reduced mechanically induced calcium increases. a-k Calcium imaging with HEK293 cells transiently transfected with the FRET constructs H_1R-ct-b (**a**) H_1R-ct-b-F484P (**b**) or H_1R-ct-b-F484P-F488P (**c**), AT_1R (**e** and **h**), AT_1R with H8 mutations AT_1R-F309P (**f** and **i**) and AT_1R-F309P-F313P (**g** and **j**). **a-c**, **e-g**, **h-j** Representative traces of $[Ca^{2+}]_i$ with application of hypoosmotic solution (Hypo) or 100 nM angiotensin II (AII). **d, k** Summaries of $[Ca^{2+}]_i$ before and during application of hypoosmotic solution (**d**) or of angiotensin II (**k**). **d** ***P<0.001, black asterisks; Wilcoxon matched-pairs signed-rank test to compare to basal $[Ca^{2+}]_i$ and ***P<0.001, magenta asterisks; Mann-Whitney U test to compare to the H_1R-ct-b construct. **k** ***P<0.001, black asterisks; Wilcoxon matched-pairs signed-rank test to compare to basal $[Ca^{2+}]_i$ before application of angiotensin II. (**d** and **k**) n = x/y indicates the sample size, where x is the number of measured cells and y is the number of coverslips from at least 3 experimental days. Data are presented as boxplots with means plus interquartile ranges. Whiskers indicate 1.5fold interquartile range. Related to Figure 4.

Supplementary Methods

Primer name	Sequence (5‘-3‘)	Length (bp)
**hAT_1R-F309P sense**	GGGGAAAAAACCCAAAAGATATTTTCTCCAGC	**32**
**hAT_1R-F309P antisense**	AGAAAGCCATAAAAAAGAGG	**20**
**hAT_1R-F309P-F313P sense**	CAAAAGATATCCCCTCCAGCTTCTAAAATATATTC	**35**
**hAT_1R-F309P-F313P antisense**	GGTTTTTTCCCCAGAAAG	**18**
**gpH_1R-D116A sense**	CTTCTGGCTCTCTATGGCTTATGTGGCCAGCACAG	**35**
**gpH_1R-D116A antisense**	CTGTGCTGGCCACATAAGCCATAGAGAGCCAGAAG	**35**
**gpH_1R-D116A-F433A sense**	TCTCTGCTGGATCCCCTACGCTGTGTTCTTCATGGTCATT	**40**
**gpH1R-D116A-F433A antisense**	AATGACCATGAAGAACACAGCGTAGGGGATCCAGCAGAGA	**40**
**gpH_1R-F484P sense**	GTGCGCGGTGCAATGAGAATCCCAGGAAGACCTTCAAGAG	**40**
**gpH_1R-F484P antisense**	CTCTTGAAGGTCTTCCTGGGATTCTCATTGCACCGCGCAC	**40**
**gpH_1R-F484P-F488P sense**	GAGAATCCCAGGAAGACCCCCAAGAGGATCCTGCGTAT	**38**
**gpH_1R-F484P-F488P antisense**	ATACGCAGGATCCTCTTGGGGGTCTTCCTGGGATTCTC	**38**
**$hA_{2A}R$-T298P sense**	GCGAGTTCCGCCAGCCCTTCCGCAAGATC	**29**
**$hA_{2A}R$-T298P antisense**	GATCTTGCGGAAGGGCTGGCGGAACTCGC	**29**
**gpH_1R^{trunc} C472Stop sense**	CACTCATCTACCCACTGTGAAATGAGAATTTCAGGAAGA	**39**
**gpH_1R^{trunc} C472Stop antisense**	TCTTCCTGAAATTCTCATTTCACAGTGGGTAGATGAGTG	**39**
**gpH_1R-ct-b-SacII sense (1.mutation)**	GAACCCACTCATCTACCCGCTGTGCAATGAGAATTTCA	**38**
**gpH_1R-ct-b-SacII antisense (1.mutation)**	TGAAATTCTCATTGCACAGCGGGTAGATGAGTGGGTTC	**38**
**gpH_1R-ct-b-SacII sense (2.mutation)**	CCACTCATCTACCCGCGGTGCAATGAGAATTTCA	**34**
**gpH_1R-ct-b-SacII antisense (2.mutation)**	TGAAATTCTCATTGCACCGCGGGTAGATGAGTGG	**34**
**hGnRHR-gpH_1R Stop sense (1.mutation)**	CTTTGATCCACTTATCTATGGATATTTTTCTCTGCCATTATGCAATGAGA ATTTCAGGAAGACCTTCAAGAGGATC	**76**
**hGnRHR-gpH_1R Stop antisense (1.mutation)**	GATCCTCTTGAAGGTCTTCCTGAAATTCTCATTGCATAATGGCAGAGAA AAATATCCATAGATAAGTGGATCAAAG	**76**
**hGnRHR-gpH_1R Stop sense (2.mutation)**	TCCTGCGTATCCCCCCTTAGGAGCATGCATCTAGAGG	**37**
**hGnRHR-gpH_1R Stop antisense (2.mutation)**	CCTCTAGATGCATGCTCCTAAGGGGGGATACGCAGGA	**37**

Supplementary Methods Table 1. Primers used for mutagenesis.

Supplementary Methods

Name	Forward primer	Reverse primer	Amplicon (nt)
**H_1R**	5'-TCC TGT GCA TTG ATC GCT AC-3'	5'-CTC GGG TCT TGG TAC GAT ACT T-3'	72
**H_2R**	5'-GAT CAG GCC AAG AGG ATC G-3'	5'-TGT CAC TGT GGC TCT GTG C-3'	74
**H_3R**	5'-AGC TAC GAC CGC TTC CTG T-3'	5'-AGC AGC ATC TTC CGC ACT-3'	89
**H_4R**	5'-GGA AGA CTA CAC ATT TTA GGT ATG TGA-3'	5'-TGA TGA AGA AGG TCA AAT TAG CAA-3'	91
**M_1R**	5'-TGT TTG GGT CCC TGG AGA-3'	5'-CTC AGG GGA AAG TCA TCA CC-3'	100
**M_3R**	5'-CAA GCT TCC GGG TCA CAG -3'	5'-AGG TTG TCC GAT GAG GGT AA-3'	60
**M_5R**	5'-CAC CAT CAC TTT TGG CAC TG-3'	5'-GTT TCC CGG TAG ATT CGA CA-3'	87
**ET_AR**	5'-ACC CCA TAG CTC TGT ATT TTG TG-3'	5'-CGA GGT CAT CAG ACT TTT GGA-3'	98
**ET_BR**	5'-ATC GTC ATT GAC ATC CCT ATC A-3'	5'-GCT TAC ACA TCT CAG CTC CAA A-3'	76
**$V_{1A}R$**	5'-TTG TGA TCG TGA CGG CTT AC-3'	5'-GAT GGT AGG GTT TTC CGA TTC-3'	100
**V_2R**	5'-CCG TGG CTC TGT TCC AAG-3'	5'-TAC TTC ACG GCC CGA CAC-3'	91
**B_1R**	5'-GCC AAC TTC TTT GCC TTC AC-3'	5'-CGG CCC ACA AAG ACA TAA AT-3'	62
**B_2R**	5'-ATG TTT CTG TCT GTT CGT GAG G-3'	5'-CTC CAC TTG GGG GCA TTT-3'	129
**$P2Y_1R$**	5'-ATG TTC TGT GTC CCC TTG GT-3'	5'-AAA TCA AAG CTC TCA CAA TTA ATC C-3'	64
**$CysLT_1R$**	5'-TGC AGA AGT CCG TGG TCA TA-3'	5'-AGG AGA GGG TCA AAG CAA CA-3'	64
**$CysLT_2R$**	5'-TCA TGG CTT CCT CAA TAA TGC-3'	5'-GAT GTG ACA CTG CCG TTC TG-3'	61
**UT_2R**	5'-GCA ACC CTC AAC AGC TCC T-3'	5'-CAG CAG AGT CCC AAT GGT G-3'	87
**$alpha_{2A}R$**	5'-ACA TCC CCA GTT GTT GGT TT-3'	5'-GGG TGG CCC ACT AGG AAG-3'	61
**$beta_2R$**	5'-CAG GAA GCC ATC AAC TGC TA-3'	5'-CAT AGG CTT GGT TCG TGA AGA-3'	64
**$beta_3R$**	5'-ACC TGG TGA TGG GAC TCC T -3'	5'-GTC CAC CGA GGT CCA CAG-3'	104
**GPR68**	5'-CAG AAG AGC CGC AAG GAC-3'	5'-AGG CCA GGA AGA TGA CCA C-3'	64
**AT_1R**	5'-CTG GCC CTT TGG CAA TTA-3'	5'-AAC ACA CTA GCG TAC AGG TTG AAA-3'	72
**PTH_1R**	5'-GGT GGA GGG GCT GTA CCT-3'	5'-ACC CAC ACA GCC ACG AAG-3'	120
**D_5R**	5'-AAC CGG GAG GTG GAC AAC-3'	5'-GGG ACG TCT GAT AGA TCT GGA-3'	70
**CD31**	5'-GCA ACA CAG TCC AGA TAG TCG T-3'	5'-GAC CTC AAA CTG GGC ATC AT-3'	74
**vWF**	5'-GAG GAC CTG CAG ATG GAC TG-3'	5'-CAT AGA CGG GGG ACA GCT T-3'	64
**Cadherin**	5'-CTC CAT GTG CCG GAT AGC-3'	5'-CGA TTT CAC CAG AAG CCT CTA C-3'	92
**Hprt1**	5'-TGA CCT TGA TTT ATT TTG CAT ACC-3'	5'-CGA GCA AGA CGT TCA GTC CT-3'	102
**Ywhaz**	5'-GAT CCC CAA TGC TTC ACA AG-3'	5'-TGC TTG TTG TGA CTG ATC GAC-3'	130
**Sdha**	5'-GGA CCT GGT TGT CTT TGG TC-3'	5'-CCA GCG TTT GGT TTA ATT GG-3'	93

Supplementary Methods Table 2. Primer used for quantitative RT-PCR with HUVEC.
