# Supplementary material for: Helix 8 is the essential structural motif of mechanosensitive GPCRs
Source: Nat Commun. 2019 Dec 19;10:5784. doi: 10.1038/s41467-019-13722-0 (PMC6923424; doi:10.1038/s41467-019-13722-0)
Supplement: Supplementary file 2 — Reporting Summary [file 41467_2019_13722_MOESM2_ESM.pdf]

nature research

Corresponding author(s): Michael Mederos y Schnitzler

Last updated by author(s): Nov 11, 2019

Reporting Summary

Nature Research wishes to improve the reproducibility of the work that we publish. This form provides structure for consistency and transparency in reporting. For further information on Nature Research policies, see Authors & Referees and the Editorial Policy Checklist.

Statistics

For all statistical analyses, confirm that the following items are present in the figure legend, table legend, main text, or Methods section.

n/a	Confirmed	
☐	☒	The exact sample size (*n*) for each experimental group/condition, given as a discrete number and unit of measurement
☐	☒	A statement on whether measurements were taken from distinct samples or whether the same sample was measured repeatedly
☐	☒	The statistical test(s) used AND whether they are one- or two-sided<br>*Only common tests should be described solely by name; describe more complex techniques in the Methods section.*
☐	☒	A description of all covariates tested
☐	☒	A description of any assumptions or corrections, such as tests of normality and adjustment for multiple comparisons
☐	☒	A full description of the statistical parameters including central tendency (e.g. means) or other basic estimates (e.g. regression coefficient) AND variation (e.g. standard deviation) or associated estimates of uncertainty (e.g. confidence intervals)
☒	☐	For null hypothesis testing, the test statistic (e.g. *F*, *t*, *r*) with confidence intervals, effect sizes, degrees of freedom and *P* value noted<br>*Give P values as exact values whenever suitable.*
☒	☐	For Bayesian analysis, information on the choice of priors and Markov chain Monte Carlo settings
☒	☐	For hierarchical and complex designs, identification of the appropriate level for tests and full reporting of outcomes
☒	☐	Estimates of effect sizes (e.g. Cohen's *d*, Pearson's *r*), indicating how they were calculated

Our web collection on statistics for biologists contains articles on many of the points above.

Software and code

Policy information about availability of computer code

Data collection	Roche LightCycler 480 and Software release 1.5.0.39, TILLPhotonics Dual Emission Photometry with Polychrom V and Patchmaster v2x90.1, HEKA EPC-10 and Patchmaster v2x90.1, DMT Pressure Myograph 111P and MyoView 1.2P, TILL-Photonics Calcium-Imaging and TILLvisION 4.5.57, Leica TCS SP5 II.
Data analysis	FitMaster v2x73.1, OriginPro 7.5, OriginPro 2019b, GraphPad Prism 7, GraphPad Prism 8

For manuscripts utilizing custom algorithms or software that are central to the research but not yet described in published literature, software must be made available to editors/reviewers. We strongly encourage code deposition in a community repository (e.g. GitHub). See the Nature Research guidelines for submitting code & software for further information.

Data

Policy information about availability of data

All manuscripts must include a data availability statement. This statement should provide the following information, where applicable:

- Accession codes, unique identifiers, or web links for publicly available datasets
- A list of figures that have associated raw data
- A description of any restrictions on data availability

Data supporting the findings of this study are available from the corresponding author upon reasonable request.
A reporting summary for this article is available as a Supplemntary Information file.
Figs. 1a, 1c, 1e-j, 2e, 2f, 2h, 2j, 2j, 2l, 2n, 3a-c, 3g, 3h, 4b-g, 4k, 4m, 5a-e, 5j and Supplementary Figs. 1c-f, 2a-d, 2l, 3k-n, 4b-e, 5a, 5b, 6d and 6k are provided as a Source Data file.

Field-specific reporting

Please select the one below that is the best fit for your research. If you are not sure, read the appropriate sections before making your selection.

☒ Life sciences ☐ Behavioural & social sciences ☐ Ecological, evolutionary & environmental sciences

For a reference copy of the document with all sections, see nature.com/documents/nr-reporting-summary-flat.pdf

Life sciences study design

All studies must disclose on these points even when the disclosure is negative.

Sample size	No sample size calculation was performed.
Data exclusions	Outliers were tested using interative Grubb's test. When P≤0.05, outliers were removed.<br>In case of flow-induced vasodilation measurements, 29 values from 2250 values (22 values in Fig 1h and 7 values in Fig 1i) were excluded during the first three and the last 5 minutes due to measurement artefacts that were caused by technical problems during the experiment.
Replication	All attempts to replicate the results were successful. Each experiment was repeated at least three times with at least three biological replicates at at least three experimental days.
Randomization	Randomization was not relevant to our study.
Blinding	Blinding was not applied in our study. The experimentors prepared the cells for analysis or performed genotyping of mice prior to experiments.

Reporting for specific materials, systems and methods

We require information from authors about some types of materials, experimental systems and methods used in many studies. Here, indicate whether each material, system or method listed is relevant to your study. If you are not sure if a list item applies to your research, read the appropriate section before selecting a response.

Materials & experimental systems

n/a	Involved in the study	
☒	☐	Antibodies
☐	☒	Eukaryotic cell lines
☒	☐	Palaeontology
☐	☒	Animals and other organisms
☒	☐	Human research participants
☒	☐	Clinical data

Methods

n/a	Involved in the study	
☒	☐	ChIP-seq
☒	☐	Flow cytometry
☒	☐	MRI-based neuroimaging

Eukaryotic cell lines

Policy information about cell lines

Cell line source(s)	HEK293 cells (Leibnitz-Institute DSMZ; 293T, DSMZ no.: ACC 635)<br>HEK293 cells (Leibnitz-Insitute DSMZ; 293, DSMZ no.: ACC 305)<br>CHO-K1 cells (Leibnitz-Insitute DSMZ; CHO-K1, DSMZ no.: ACC-110)<br>human umbilical vein endothelial cells (Promocell; HUVEC, C-12259)
Authentication	The cell lines were bought from Leibnitz-Institute DSMZ or from Promocell.
Mycoplasma contamination	All cell lines were regularly tested negative for myocoplamsa contamination.
Commonly misidentified lines (See ICLAC register)	N/A

Animals and other organisms

Policy information about studies involving animals; ARRIVE guidelines recommended for reporting animal research

Laboratory animals	Wildtype C57BL/6J mice were from Janvier.<br>H1R gene-deficient mice were from Takeshi Watanabe and Perti Panula (PMID: 8917588).<br>H1/2/3/4R quadruple gene-deficient mice were from Cory Teuscher (PMCID: PMC3747232).<br>tamoxifen-inducible, smooth muscle specific Gq/11-protein knock-down mice were from Stefan Offermanns and Angela Wirth

	(PMID: 18084302).<br>All animals used in the study were male and 10-15 weeks old.
Wild animals	N/A
Field-collected samples	N/A
Ethics oversight	All animal studies were approved and overseen by the governmental oversight authority District Government of Upper Bavaria (Germany).

Note that full information on the approval of the study protocol must also be provided in the manuscript.
